# SnowyOwl: accurate prediction of fungal genes by using RNA-Seq and homology information to select among ab initio models

**DOI:** 10.1186/1471-2105-15-229

**Published:** 2014-07-01

**Authors:** Ian Reid, Nicholas O’Toole, Omar Zabaneh, Reza Nourzadeh, Mahmoud Dahdouli, Mostafa Abdellateef, Paul MK Gordon, Jung Soh, Gregory Butler, Christoph W Sensen, Adrian Tsang

**Affiliations:** 1 Centre for Structural and Functional Genomics, Concordia University, 7141 Sherbrooke St. W, Montreal, QC H4B 1R6, Canada; 2 Faculty of Medicine, Visual Genomics Centre, University of Calgary, 3330 Hospital Drive NW, Calgary, AB T2N 4N1, Canada

**Keywords:** RNA-Seq, Gene prediction, Fungi, *Aspergillus niger*, *Phanerochaete chrysosporium*, *Thermomyces lanuginosus*, *Neurospora crassa*

## Abstract

**Background:**

Locating the protein-coding genes in novel genomes is essential to understanding and exploiting the genomic information but it is still difficult to accurately predict all the genes. The recent availability of detailed information about transcript structure from high-throughput sequencing of messenger RNA (RNA-Seq) delineates many expressed genes and promises increased accuracy in gene prediction. Computational gene predictors have been intensively developed for and tested in well-studied animal genomes. Hundreds of fungal genomes are now or will soon be sequenced. The differences of fungal genomes from animal genomes and the phylogenetic sparsity of well-studied fungi call for gene-prediction tools tailored to them.

**Results:**

SnowyOwl is a new gene prediction pipeline that uses RNA-Seq data to train and provide hints for the generation of Hidden Markov Model (HMM)-based gene predictions and to evaluate the resulting models. The pipeline has been developed and streamlined by comparing its predictions to manually curated gene models in three fungal genomes and validated against the high-quality gene annotation of *Neurospora crassa*; SnowyOwl predicted *N. crassa* genes with 83% sensitivity and 65% specificity. SnowyOwl gains sensitivity by repeatedly running the HMM gene predictor Augustus with varied input parameters and selectivity by choosing the models with best homology to known proteins and best agreement with the RNA-Seq data.

**Conclusions:**

SnowyOwl efficiently uses RNA-Seq data to produce accurate gene models in both well-studied and novel fungal genomes. The source code for the SnowyOwl pipeline (in Python) and a web interface (in PHP) is freely available from http://sourceforge.net/projects/snowyowl/.

## Background

A prime motivation for determining the genomic sequence of an organism is to obtain information about the genes it contains and the proteins they encode. Accurate prediction and characterization of gene structures enable genome-wide analyses including the identification of target genes, transcriptome and proteome studies, and comparative genomics and evolutionary analyses. As DNA sequencing technology advances and whole genome sequences accumulate, gene prediction has become a bottleneck, especially in newly-sequenced genomes.

Gene prediction software has been developed intensively over the last fifteen years; numerous programs are already available [1] and more continue to appear. These programs commonly use three basic sources of evidence, alone or in combination. Genes can be predicted “ab initio” by detecting statistical signals of gene structure in DNA sequences using Hidden Markov Models (HMMs) [2-6], Conditional Random Fields [7], and Support Vector Machines [8]. Genes can also be predicted by sequence homology with known genes or proteins in related organisms [9,10], or with protein family profiles [11]. Experimental evidence from the target organism, such as protein, EST, or transcript sequences, can help identify individual genes [12,13]. The most successful gene predictors have integrated all available evidence, either internally [12] or by combining the predictions of other programs [14].

The massively parallel sequencing of short fragments of messenger RNA, RNA-Seq, can yield detailed information on the structure of many of the mature transcripts in an organism [15]. The short sequences from RNA-Seq can be combined to delineate transcripts by assembly into contigs [16,17] or by mapping to the genome sequence [18]. The arrival of this source of experimental evidence has stimulated the development of a new generation of computational tools to apply it to gene and transcript prediction and quantification [19].

The effectiveness of different gene prediction programs in various animal genomes have been compared in a series of competitions: GASP for the fruit fly genome [20], EGASP for the human genome [21], and nGASP for the nematode genome [22]. The competition organizers chose genome sections with high-quality gene annotations as a test set, invited program authors to predict the genes in the test set from their DNA sequences, and compared the predictions to the annotated genes. Gene prediction sensitivity was estimated by the fraction of annotated genes that were predicted, and gene prediction specificity by the fraction of predictions that matched annotated genes. Sensitivity and specificity were also assessed at transcript, exon, and nucleotide levels. Recently the RGASP consortium has used a similar framework to test programs that reconstruct transcripts from RNA-Seq reads in the human, fly, and nematode genomes [19].

The genomes of hundreds of fungi have been sequenced recently or are being sequenced currently in large projects such as 1000 Fungal Genomes [23] and the Fungal Genome Initiative [24], and in smaller studies. This effort is motivated by curiosity about the genetic diversity and phylogeny of fungi, by their ecological importance, and by their economic impact as pathogens of plants and animals, including humans, and as sources of food, medicines, industrial chemicals and enzymes.

Development of eukaryotic gene prediction software has mainly targeted animal genomes; the resulting tools have then been applied to fungi, plants, and other biological groups. Fungal genomes show several differences from typical animal genomes: they are more compact, with shorter intergenic spaces and introns [25]. Gene spacing may be so tight that the untranslated regions (UTRs) of adjacent genes overlap. Sequences that signal transcription start and stop and translation start sites in animal genomes are not active in fungal genomes, and their equivalents in fungi are not well understood [26]. Despite the recent activity, a very small fraction of fungal species have been sequenced, and even fewer have well-characterized genes and gene products; this currently limits the possibility of detailed comparative gene prediction to a few well-studied genera. Manual curation of gene predictions cannot keep up with the pace of fungal genome sequencing and computational gene prediction [27]. Many predicted fungal proteins in the sequence databases lack experimental verification. In addition, many of the newly-sequenced genomes are assembled solely from short reads, which is still challenging and increases chances for assembly errors [28]. Genome sequence errors that introduce reading frame shifts or that add or remove start or stop codons or intron donors or acceptors can lead to major errors in gene prediction. Fragmented assemblies can cut gene sequences in two. These characteristics of fungal genomes can best be accommodated in a gene predictor that is tailored to them.

In the Genozymes project [29] we are analyzing the newly-sequenced genomes of 26 thermophilic fungi in search of novel and useful enzymes. We needed a quick and accurate method to identify the genes in these genomes and predict the sequences of their proteins. The genome assemblies and RNA-Seq reads are available but other resources such as EST collections or close relatives with annotated genomes usually are not. We have developed an efficient fungal gene prediction pipeline, SnowyOwl, that makes extensive use of RNA-Seq data. SnowyOwl generates initial models for training an HMM gene predictor by assembling RNA-Seq reads into predicted transcripts, and additionally uses the intron and transcribed sequence positions revealed by mapping RNA-Seq reads onto the genome assembly to guide and evaluate gene predictions. The SnowyOwl source code is publicly available and the pipeline is also available as a web service.

To demonstrate the effectiveness of SnowyOwl we have predicted genes in the genomes of the basidiomycete *Phanerochaete chrysosporium* and the ascomycetes *Aspergillus niger* and *Thermomyces lanuginosus*, and compared them with our manually curated gene sets as well as previous annotations of these genomes. The SnowyOwl predictions are more sensitive and accurate in these comparisons than published gene models for *A. niger*[30] and *P. chrysosporium*[31]. We also validated the performance of SnowyOwl on an independently annotated reference genome, in the manner of GASP, EGASP, nGASP, and RGASP. This required a filamentous fungus with high-quality gene annotations. The best-studied fungal genome is that of the yeast *Saccharomyces cerevisiae*[32], but it differs significantly from the genomes of filamentous fungi [25]. *Neurospora crassa* is a better standard for our purpose. It was the first filamentous fungus to have its genome sequenced [33] and its sequence has been upgraded to finished status [34]. The structural and functional annotation of its genes has undergone several revisions, most recently with the aid of Roche 454-sequenced transcripts and strand-specific RNA-Seq reads [35]. The reliability of the *N. crassa* annotations may not be as unassailable as those used in the GASP contests, but we believe that it is sufficient for a useful benchmark.

## Implementation

### Overview

**Figure 1:**
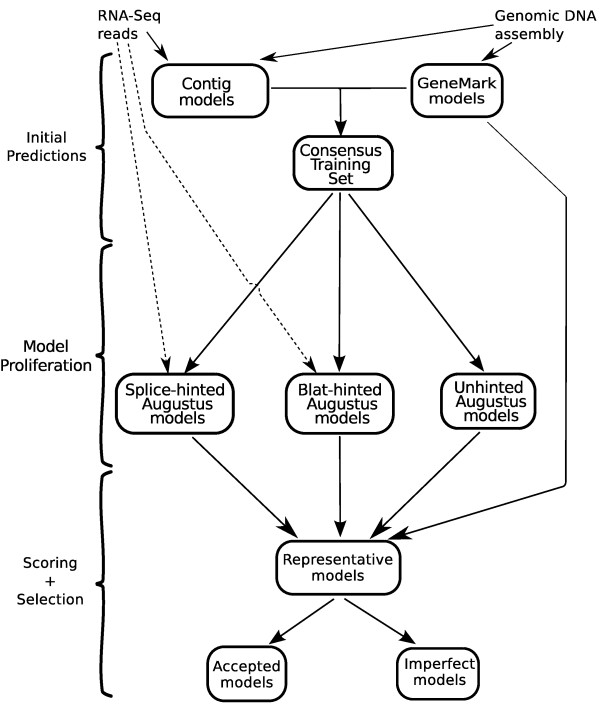
Stages of information flow from DNA and RNA sequences to gene models in the SnowyOwl pipeline.

The SnowyOwl pipeline takes as inputs a genome sequence file, a file of RNA-Seq reads, a file of full-length transcript sequences assembled from the RNA-Seq reads, a file of intron locations, and a file of RNA-Seq read coverage values [see Additional file 1]. The RNA-Seq reads do not need to be strand-specific. First a pool of candidate gene models is generated with the HMM-based gene predictors Augustus and GeneMark-ES, then the models are scored against the RNA-Seq intron and coverage evidence, and finally the set of non-overlapping models with the highest total score is selected (Figure 1). SnowyOwl does not attempt to predict alternative splicing isoforms, which is difficult even in well-studied genomes [19]. Each transcript predicted by Augustus is treated as a separate gene candidate and only the best-supported model at each location is retained during selection. Nor does SnowyOwl attempt to predict UTRs; the gene prediction includes only the coding sequence (CDS).

The main product of the pipeline is a set of **accepted** gene models. Accepted models conform to the general principles of fungal exon and intron structure, include an open reading frame (ORF) that encodes a protein of reasonable length, and are consistent with RNA-Seq read coverage distribution and the introns implied by spliced reads if their read coverage is adequate. In addition to the accepted models, the pipeline produces a number of **imperfect** models from genomic regions with no accepted model. While these models fail to meet one or more of the criteria for acceptability, they serve as reminders that some evidence exists for genes at their locations. The highest scoring of the models, accepted or imperfect, predicted at any location is selected as a **representative**.

### Candidate generation

Augustus [2,11,12] was among the most sensitive gene and transcript predictors in the nGASP [22] and RGASP [19] contests, accepts a broad range of evidence sources, and has been used successfully with several fungal genomes. GeneMark-ES has the virtue of being self-training, and has also been used successfully in fungal genomes [4].Augustus requires a training set of at least several hundred gene models from the target genome. SnowyOwl creates this training set in a bootstrap fashion. Augustus is first trained on the input transcript sequences and run to generate a set of initial gene models, termed Contig models (Figure 1). A second set of initial models is obtained by running GeneMark-ES. To increase its reliability, the Consensus Training Set is formed from models that are common to the Contig models and the GeneMark models. The Consensus Training Set is used to retrain Augustus for a second round of predictions.

In this Model Proliferation stage, the criteria for model acceptance by Augustus are relaxed to increase the diversity of the gene models generated. By default Augustus reports a single, best prediction at a location; it can, however, be configured to generate alternative transcripts from probabilistic sampling or from evidence provided by hints, with the number of alternatives controlled by thresholds for the minimum and mean posterior probabilities of exons and introns in the models. Often the model selected by the SnowyOwl pipeline was not the alternative that Augustus predicted to be most probable, so we prefer to have Augustus produce a wide variety of alternatives for scoring and selection by the pipeline.

Hints based on RNA-Seq data are used to guide the Augustus predictions. One set of hints is generated from the input files of intron locations and RNA-Seq read coverage, leading to the Splice-hinted Augustus models. Another hint set is derived by mapping the RNA-Seq reads to the genome with Blat as described in the Augustus documentation [12]. Hints provided to Augustus are weighted and evaluated according to parameters from an extrinsic configuration file; the SnowyOwl pipeline runs Augustus with seven different extrinsic configuration files for the RNA-Seq hints to increase the number of alternative models generated. The union of all the Blat-hinted Augustus and unhinted Augustus prediction sets forms the Pooled Augustus Models.

### Scoring of gene models

**Figure 2:**
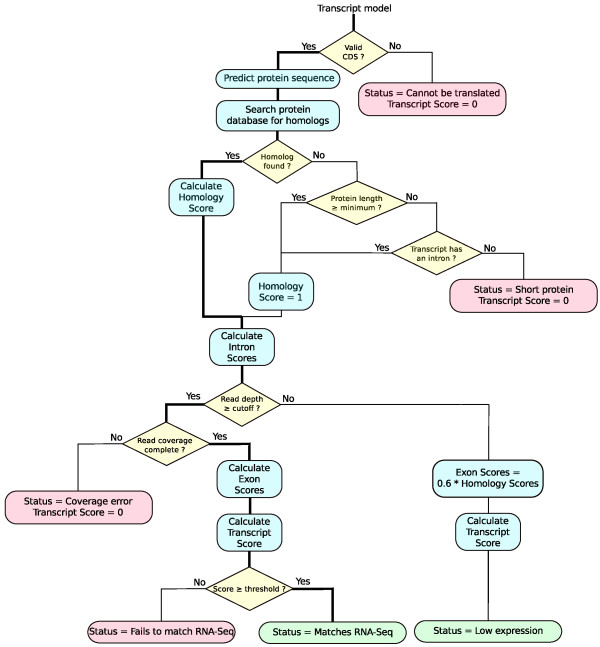
**Model scoring flowchart.** The bold line marks the main path. Models that follow paths leading to boxes with pink backgrounds are imperfect; models with paths ending in boxes with green backgrounds are potentially accepted.

At the heart of the SnowyOwl pipeline is the selection of the best available gene model at each location within the genome. ‘Best’ is operationally defined through a model-scoring procedure summarized in Figure 2 and the following formulas. Since each SnowyOwl gene model has a unique transcript the transcript score *V*_*t*_(*t*) serves as the gene score. Scoring depends on several configurable parameters [see Additional file 2]; Table 1 shows the parameter values that we used to obtain the results reported here. Dealing with special circumstances such as low read coverage, short predicted proteins, and partial intron retention complicates the scoring logic (Figure 2).

**Table 1:** Adjustable parameters for model scoring and read island assembly

**Parameter**	**Default value**
Maximum homolog count	3
Acceptable intron donor-acceptor pairs	GT-AG, GC-AG, AT-AC
Minimum intron length	10
Maximum intron length	2000
Minimum protein length	150 amino acids
Low median coverage cutoff	10
Low coverage penalty factor	0.6
Acceptable range of exon coverage depth	0.15 to 3 × transcript median depth
Maximum length of low coverage run	10
Low coverage run threshold	0.05 × transcript mean depth
Maximum N count per transcript	8
Threshold score for “Matches RNA-Seq data” status	0.5
Selection pseudoscore	0.0001
Read island minimum cover depth	3
Read island minimum length	100

Scoring begins by translating the predicted transcripts into proteins and using BLASTp [36] with the default BLOSUM62 substitution matrix to search for homologs in a protein database. The sequences of RefSeq proteins from fungi were downloaded from NCBI [37] on March 14, 2010. The Uniprot-Swissprot database was downloaded from [38] in November 2010. Any model without a valid coding sequence is assigned a score of 0 and not considered further. A homology score is calculated for each significant BLASTp hit *B*_*i*_ from its fraction of matching and similar amino acids as reported by BLASTp, up to a maximum number of hits, and divided among the transcript exons *e* according to their overlap with the hit.

Homology score:

$$\begin{array}{c} H_{i}\left(t\right)=\frac{1}{\text{maxhomologs}}\cdot \frac{\text{Matches}\left(B_{i}\right)+\text{Similarities}\left(B_{i}\right)}{2\text{Len}\left(B_{i}\right)}\\ \;H\left(e\right)=1+\sum_{i=1}^{\text{maxhomologs}}H_{i}\left(t\right)\cdot \frac{\text{Len}\left(e\cap B_{i}\right)}{\text{Len}\left(e\right)} \end{array}$$

Predicted proteins shorter than a minimum length lead to a score of 0 unless the transcript model contains an intron or the protein has at least one BLASTp hit.

Each predicted intron is scored 0 if it uses unconventional donor-acceptor sequences or if it is longer or shorter than set limits.

Intron score:

$$I\left(i\right)=\left\{\begin{array}{c} 0\;\mathrm{if}\;\text{donor}‒\text{acceptor}\;\notin \left(\mathrm{GT}‒\mathrm{AG},\;\mathrm{GC}‒\mathrm{AG},\;\mathrm{AT}‒\mathrm{AC}\right)\\ 0\;\mathrm{if}\;\text{length}\left(i\right)<\;\text{minimum}\\ 0\;\mathrm{if}\;\text{length}\left(i\right)>\text{maximum}\\ 1\;\text{otherwise} \end{array}\right\}$$

Models with RNA-Seq read coverage too low for reliable assessment of coverage continuity or splice junction usage bypass detailed scoring and receive a final score based only on their homology and intron scores. Models with adequate median coverage are rejected if the coverage is not continuous over their whole length.

Each exon in well-covered model transcripts is scored for agreement with the splice junctions in the RNA-Seq reads. Exon ends matching a splice junction receive a bonus, and ends that miss a splice junction incur a penalty. Exons that cover splice junctions are also penalized.

Exon score:

$$V_{x}\left(e\right)=H\left(e\right)\cdot R_{\text{left}}\left(e\right)\cdot R_{\text{mid}}\left(e\right)\cdot R_{\text{right}}\left(e\right)$$

Exon boundary scores:

$$R_{\text{left}}\left(e\right)=\left\{\begin{array}{c} 1\;\mathrm{if}\;e\;\mathrm{is}\;\mathrm{an}\;\mathrm{initial}\;\mathrm{exon}\\ \text{Reward}\left(j\right)\;\mathrm{if}\;\text{the}\;\text{start}\;\mathrm{of}\;e\;\text{matches}\;\text{the}\;\text{end}\;\mathrm{of}\;\text{splice}\;\text{junction}\;j\\ \text{Penalty}\left(j\right)\;\mathrm{if}\;\text{the}\;\text{start}\;\mathrm{of}\;e\;\text{does}\;\text{not}\;\text{match}\;\text{the}\;\text{nearest}\;\text{splice}\;\text{junction}\;j\\ 0\;\mathrm{if}\;\text{there}\;\mathrm{is}\;\mathrm{no}\;\text{splice}\;\text{junction}\;\text{between}\;\text{the}\;\text{transcript}\;\text{start}\;\text{and}\;\text{the}\;\text{end}\;\mathrm{of}\;e \end{array}\right\}$$

$$R_{\text{mid}}\left(e\right)=\left\{\begin{array}{c} 1\;\text{if}\;e\;\text{does}\;\text{not}\;\text{completely}\;\text{contain}\;\text{any}\;\text{splice}\;\text{junction}\\ \text{Penalty}\left(j\right)\;\mathrm{if}\;e\;\text{completely}\;\text{contains}\;\text{splice}\;\text{junction}\;j \end{array}\right\}$$

$$R_{\text{right}}\left(e\right)=\left\{\begin{array}{c} 1\;\mathrm{if}\;e\;\mathrm{is}\;a\;\text{terminal}\;\text{exon}\\ \text{Reward}\left(j\right)\;\mathrm{if}\;\text{the}\;\text{end}\;\mathrm{of}e\text{matches}\;\text{the}\;\text{start}\;\mathrm{of}\;\text{splice}\;\text{junction}\;j\\ \text{Penalty}\left(j\right)\;\mathrm{if}\;\text{the}\;\text{end}\;\mathrm{of}e\text{does}\;\text{not}\;\text{match}\;\text{the}\;\text{start}\;\mathrm{of}\;\text{the}\;\text{nearest}\;\text{splice}\;\text{junction}\;j\\ 0\;\mathrm{if}\;\text{there}\;\mathrm{is}\;\mathrm{no}\;\text{splice}\;\text{junction}\;\text{between}\;\text{the}\;\text{start}\;\mathrm{of}\;e\;\text{and}\;\text{the}\;\text{transcript}\;\text{end} \end{array}\right\}$$

Introns are sometimes retained in spliced transcripts, and each splice junction has an empirical read-through ratio to reflect this.

Read-through ratio:

$$\mathrm{RT}\left(j\right)=\;\frac{\;\text{mean}\;\text{read}\;\text{depth}\;\text{inside}\;\text{junction}\;j}{\text{mean}\;\text{read}\;\text{depth}\;\text{inside}\;\text{junction}\;j+\text{number}\;\mathrm{of}\;\text{spliced}\;\text{reads}\;\text{spanning}\;\text{junction}\;j}$$

The splice junction bonuses and penalties are functions of the read-through ratio.

$$\begin{array}{c} \text{Reward}\left(j\right)=2\sqrt{1-\mathrm{RT}\left(j\right)}\\ \text{Penalty}\left(j\right)=1-\sqrt{1-\mathrm{RT}\left(j\right)} \end{array}$$

The transcript score is the product of the scores of its introns and introns.

Transcript score:

$$V_{t}\left(t\right)=\prod_{i\in \;\text{Introns}\left(t\right)}I\left(i\right)\;•\prod_{e\in \;\text{Exons}\left(t\right)}V_{x}\left(e\right)$$

The scored models are classified by their final score into those that match the RNA-Seq data and those that do not (Figure 2).

### Selection of representative models

**Figure 3:**
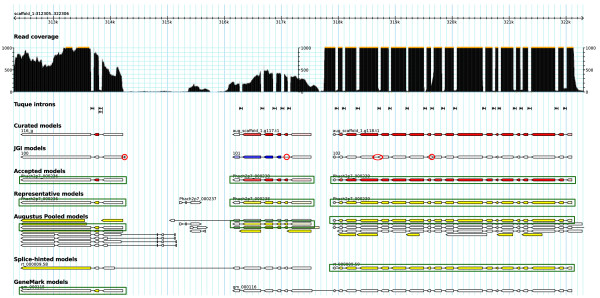
**Selection of the best scored models in a typical region of the *P. chrysosporium *genome with overlapping gene predictions.** Representative models are the highest scoring models at their locations, and accepted models are representatives that are consistent with all available evidence. In this region, the SnowyOwl accepted models matched the manually curated models, but previous (JGI) models showed small differences (marked with red ovals). Accepted models and the representative and candidate models that match them are outlined in green. The colour intensity in each exon is proportional to its score. Marked introns were verified by detecting spanning spliced reads with tuqueSplit [39]. Orange bars at the top of the read coverage track show regions of coverage depth > 1000. The data were visualized with GBrowse2 [40].

The selection routine acts on sets of scored gene models sorted by start position. Models that overlap one another are grouped together. Within each group, the chains of non-overlapping model transcripts with the highest score summed over the member transcripts are found by dynamic programming. Transcripts with score 0 are included in the chains where there is no overlapping transcript of higher score. Regions containing multiple overlapping models that all score 0 are frequent, and could inflate the number of highest-scoring chains if all the possible alternatives were retained. To avoid such inflation and to favour the longest among 0-scoring models, a small positive per-base pseudoscore is added to the score of all models before dynamic programming. The selection routine returns the gene models in the highest-scoring chain(s).In SnowyOwl selection is applied first to the Pooled Augustus Model set, which often contains multiple predictions at the same locations. It is helpful to reduce these predictions to one or two per gene locus to avoid overwhelming the main selection with too many possible model combinations. The inputs to the main selection are the GeneMark, Splice-hinted Augustus, and preselected Pooled Augustus models. In a final pass through the genome, any model overlaps in the selected set are resolved by keeping only the highest-scoring model. If multiple models have the same score, one of them is chosen randomly. The output of this step is the representative model set. Figure 3 illustrates the selection process for one group of overlapping gene models.

The representative models are subdivided into accepted models and imperfect models, which have structural flaws or conflict with the RNA-Seq data.

## Results

### Pipeline evaluation against *Neuropora crassa* annotated models

We ran SnowyOwl on the *N. crassa* genome and compared its 10,852 accepted predictions to the latest release of annotated models from the Broad Institute, using the comparison methodology and software of the RGASP project [41]. The finished genome assembly of *Neurospora crassa* OR74A (neurospora_crassa_or74a_12_supercontigs.fasta), version 12 transcript models (neurospora_crassa_or74a_12_transcripts.gtf), and a high-confidence subset of the transcript models (Neurospora_crassa.high_confidence_gene_models.gtf) were downloaded from the Broad Institute website [34]. The RNA-Seq reads used by SnowyOwl came from a different source than those used in the selection of the annotated models and were not strand-specific. RNA-Seq reads were downloaded from the NCBI Short Read Archive [42] (accession numbers SRR627936, SRR627939, SRR627942, SRR627945, SRR627948); these reads had been generated for a study of the regulation of polysaccharide-degrading enzymes by *N. crassa*[43]. The reads were assembled into transcript sequences with Trinity [17,44] using the CuffFly option for input to SnowyOwl. To find splice junctions the reads were mapped to the *N. crassa* genome with STAR [45]. To determine read coverage the reads were mapped again with STAR using the filtered splice junctions from the first run. Read coverage depths for gene models were calculated by counting the reads mapping within the exons of each model and dividing by the total exon length. The gene models were sorted by coverage depth and subdivided at depths of 0.05, 0.1, 0.5, 1.0, 2.0, and 4.0.

**Figure 4:**
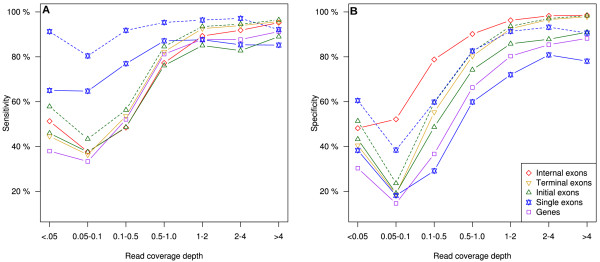
**Effect of read coverage depth on sensitivity (A) and specificity (B) of *Neurospora crassa *gene and exon prediction by SnowyOwl.** Solid lines: all coordinates matched exactly; dashed lines: exon start coordinates were not required to match. Read coverage depth is the number of reads mapped to a feature divided by the feature length.

Gene model comparisons were limited to the coding sequences. Only predictions that exactly matched an annotated model were counted as true positives (RGASP’s fixed mode). To measure prediction sensitivity, the SnowyOwl models were compared to the 8208 high-confidence *N. crassa* models; to measure prediction specificity, the SnowyOwl models were compared to all 9730 annotated models. SnowyOwl predicted 80.6% of the high-confidence annotated genes exactly and 65.3% of the SnowyOwl predictions matched an annotated gene exactly. At the exon level, SnowyOwl’s sensitivity was 82.8% and its specificity was 77.3%.The sensitivity and specificity of SnowyOwl’s predictions depended strongly on the read coverage depth (Figure 4). The sensitivity for genes and initial, internal, and terminal exons followed a common pattern: low below a depth of 0.5 and high above depth 0.5. Prediction sensitivity for monoexonic genes varied less with read depth. Differences in sensitivity between terminal and internal exons can be ascribed to stop codon prediction, and differences between initial and internal exons to start codon prediction. The generally lower sensitivity for initial exons at read depths above 1 suggests problems with placing start codons; the increased apparent sensitivity when the initial exon start is not required to match exactly (RGASP’s flexible mode) confirms this. Monoexonic genes showed a similar increase in apparent sensitivity with flexible evaluation. Prediction specificity depended even more strongly on read depth than did sensitivity. This indicates that SnowyOwl needs adequate read coverage to weed out erroneous models. The generally higher prediction specificity for internal exons than for initial, terminal, or single exons suggests that many of SnowyOwl’s wrong predictions had only one or two exons. The increased apparent specificity in flexible mode indicates frequent errors at the start codon. Among the models with RNA-Seq read coverage depth of 0.5 or more, the sensitivity of the SnowyOwl gene predictions was 88.4% and the specificity was 82.2%. If differences in start codon position were overlooked, the SnowyOwl predictions had apparent sensitivity of 93.9% and apparent specificity of 88.4% for genes with read coverage depth of 0.5 or more.

**Figure 5:**
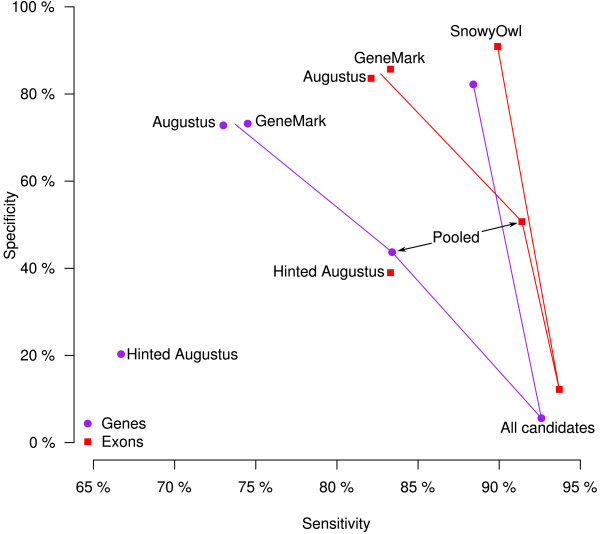
**Relationships between the sensitivity and specificity of predicting *Neurospora crassa *exons and genes by various methods.** Prediction sets were from GeneMark-ES, Augustus run with the neuropora_crassa species parameters included in the Augustus distribution, unhinted or with RNA-Seq hits, the Pooled Blat-hinted Augustus models from SnowyOwl, all the candidate models generated by SnowyOwl, and the final SnowyOwl accepted models. Models with read coverage below 0.5 were removed from each set.

Figure 5 shows how the sensitivity and specificity of *N. crassa* gene and exon predictions evolve along the SnowyOwl pipeline. GeneMark and unhinted Augustus predictions have good specificity but relatively low sensitivity. Running the hinted Augustus stage of SnowyOwl adds both correct and erroneous predictions, increasing sensitivity but lowering specificity in the pooled models. Adding the other SnowyOwl candidate models further increases sensitivity and lowers specificity. After the SnowyOwl scoring and selection stage, the specificity of the accepted models is slightly above the level of the GeneMark and Augustus predictions and their sensitivity is significantly higher. Gene and exon predictions follow roughly parallel trajectories; the sensitivity and specificity of exon predictions are always higher than those of gene predictions. Surprisingly, running Augustus with RNA-Seq hints as recommended in the Augustus documentation lowered the sensitivity as well as the specificity of gene prediction; this was apparently due to extension of predicted coding regions into the UTRs. An attempt to reduce this problem by turning on UTR prediction in Augustus was stymied by lack of UTR training data.

### Pipeline performance on manually curated models

During development of the SnowyOwl pipeline we monitored its performance by comparing its predictions to manually curated gene models from three fungal genomes. The v4.0 genome sequence of *Aspergillus niger* ATCC 1015 and filtered gene models (Aspni7_GeneCatalog_genes_20131226.gff.gz) [30] were downloaded from the Joint Genome Institute (JGI) website [46]. The v2.0 genome assembly of *Phanerochaete chrysosporium* RP78 and gene models (Pchrysosporium_BestModelsv2.1.gff.gz) [31] were also downloaded from the JGI website [46]. The genome of *Thermomyces lanuginosus* ATCC 200065 was sequenced and assembled at the McGill University Genome Quebec Innovation Centre (MUGQIC); the assembled sequence is available from [29].

Total RNA was extracted from fungi cultured on agricultural straws [47], then used to prepare mRNA-Seq libraries. Sequencing and library construction were conducted at MUGQIC; none of the libraries were strand-specific. The *A. niger* and *T. lanuginosus* libraries were sequenced to lengths of 108 bases on an Illumina GAII DNA Sequencer, while the *P. chrysosporium* library was sequenced on an Illumina HiSeq machine in a 100-base, pair-end format. Additional RNA samples from *A. niger* were sequenced to 50 bases on a HiSeq instrument. Out of the 173 million reads obtained from *A. niger*, 163 million (94%) mapped to the genome sequence, revealing 31,716 intron splice junctions. Of the 29.7 million reads from *T. lanuginosus*, 23.8 million (80%) mapped to the genome, revealing 24,691 splice junctions. A total of 454 million (91%) of the 502 million *P. chrysosporium* reads mapped to the genome, revealing 106,111 splice junctions. The read sequences have been deposited in the NCBI Short Read Archive [42] (accession numbers SRR867733-4, SRR867741, SRR867745, SRR867769-70, SRR867772, SRR867789-810, SRR867814, SRR868657-8, SRR868663, SRR868665, SRR868681, SRR945950, SRR946591).

One of the authors (AT) identified about 2000 putatively correct gene models in each of the *A. niger*, *P. chrysosporium*, and *T. lanuginosus* genomes by comparing published gene models [30,31] and predictions of GeneMark-ES and Augustus to the profiles of RNA-Seq read coverage visualized in GBrowse [40]. The selected models were computationally screened for structural validity and compatibility with the RNA-Seq evidence. This check uncovered subtle problems such as non-canonical donor-acceptor sequences in predicted introns, small shifts in intron position or the omission of short exons in some models; such erroneous models were excluded from the curated sets. The number of curated models used for benchmarking was 1855 for *P. chrysosporium*, 2055 for *A. niger*, and 2038 for *T. lanuginosus*. The curated gene model sets are available as additional files [see Additional files 3, 4, and 5].

For evaluation, the genomic coordinates of the coding sequences of a gene model were compared to a curated model exon-by-exon. Exact identity of all coordinates was required. UTR sequences were ignored. More than 93% of the curated models in *A. niger* and *T. lanuginosus* and 87% of the *P. chrysosporium* models were correctly predicted by the SnowyOwl pipeline (see Table 2). For many of the curated models that were not predicted exactly, SnowyOwl predicted a gene that differed only in the position of its start codon. Only 72% of the *A. niger* curated models and 32% of the *P. chrysosporium* curated models were included in published prediction sets [30,31].

**Table 2:** Effects of pipeline composition on number of gene predictions and recovery of curated models

		**Components in the test pipelines (leftmost is the standard SnowyOwl pipeline)**											
In Consensus Training Set	Orthologs		*		*	*	*	*	*	*	*		
	Contig Models	*	*	*		*	*	*	*	*	*	*	*
	GeneMark Models	*	*	*	*		*	*	*	*	*	*	*
In Pooled Augustus Models	Blat-hinted Models	*	*	*	*	*		*	*	*	*	*	*
	Unhinted Models	*	*	*	*	*	*		*	*	*	*	*
	Augustus Domains		*	*	*	*	*	*		*	*		
Merged with Pooled Augustus	GeneMark Models	*	*	*	*	*	*	*	*		*	*	
	Splice-hinted Models	*	*	*	*	*	*	*	*	*			
		**Changes in the number of predicted models relative to the standard pipeline**											
*A. niger*	Representative (21479)	0	6	61	-8	-21	-9332	12	-61	-27	13	23	-80
	Accepted (11374)	0	24	54	46	15	-1089	20	-35	-60	-5	-21	-149
*T. lanuginosus*	Representative (12915)	0	5	*-25*	-21	-45	-5367	6	-16	11	5	2	-11
	Accepted (7324)	0	28	13	17	0	-606	26	-7	0	14	-19	-64
*P. chrysosporium*	Representative (20201)	0	92	13	-5742	81	-6444	87	44	52	-56		
	Accepted (12669)	0	111	43	-1585	43	-2058	98	48	48	-87		
		**Recovery of curated models (%)**											
*A. niger*	Representative	94.7	94.7	94.8	94.8	94.8	94.3	94.7	94.8	91.4	91.6	91.6	91.2
	Accepted	94.5	94.5	94.6	94.5	94.5	93.4	94.5	94.5	91.2	91.3	91.3	91.0
*T. lanuginosus*	Representative	93.7	93.8	93.5	93.5	93.7	91.9	93.9	94.0	92.2	92.2	92.3	92.1
	Accepted	93.6	93.7	93.4	93.4	93.6	91.6	93.8	93.9	92.1	92.1	92.2	92.0
*P. chrysosporium*	Representative	88.0	88.1	88.0	90.4	87.8	90.9	88.2	88.2	88.2	84.6		
	Accepted	87.3	87.3	87.1	88.4	87.1	88.5	87.3	87.4	87.4	83.8		

*Component included in pipeline.

### Selection of pipeline components

While some redundancy in predictions from different sources increases confidence in those predictions, excessive redundancy increases the computational cost of operating a gene model prediction pipeline. We sought to create the lowest-cost pipeline that would accurately predict as many of the genes in a genome as possible. To find this balance, we began with a pipeline that included three sources of initial gene predictions and three types of Augustus predictions feeding into the Pooled Augustus Models. The Orthologs Training set and the Augustus Domains models, which we do not use in the final pipeline, are described in Additional file 6. To determine the contribution of each component, we left out one component of the pipeline at a time and observed the effect on prediction of the curated genes in our three test genomes. The most expensive components that were producing redundant results were iteratively dropped from the pipeline until any further reduction would have decreased sensitivity.

Table 2 presents the results of running the Snowy Owl pipeline with various components added or omitted on our three test genomes. The leftmost column represents the standard pipeline (Figure 1); columns labelled Changes show differences from this standard. In comparison to a pipeline including all components, omitting any one of Orthologs, Contig Models, or GeneMark Models from the Consensus Training Set had little effect on the number of accepted models or recovery of curated models in *A. niger* and *T. lanuginosus*. Omitting Contig models caused significant reductions in model yield in *P. chrysosporium*, however. Because Orthologs was the most computationally expensive component and was redundant in all 3 genomes, it was left out of the final pipeline.

Omitting Augustus Domains or Unhinted Augustus models from the pooled Augustus models had negligible or positive effects in all the test genomes. The multiple Augustus runs required for Augustus Domains made it the most expensive pipeline component after Orthologs, so we did not retain it. On the other hand, omitting Blat-hinted Augustus models had major negative effects on the numbers of accepted models and the recovery of curated models among the Augustus Pooled models (data not shown). Although leaving it out would have the positive effect of reducing the number of imperfect models, especially short models and models that contradicted RNA-Seq evidence, retaining this component is essential for high accepted model yield.

Omitting Splice-hinted Augustus models or GeneMark models from the selection step decreased the number of accepted models and the recovery of curated models in the representative and accepted sets in at least one genome. Consequently we kept these components in the pipeline.

### Model scoring and selection

The method of scoring and selecting models is the most original aspect of SnowyOwl. The configurable parameter values used in SnowyOwl (Table 1) were tuned to give good sensitivity on the curated models from A. niger; their general applicability was then checked on our curated models of *P. chrysosporium* and *T. lanuginosus* and the annotated models of *N. crassa*. Thus they are useful for ascomycetes and basidiomycetes, and possibly other fungi, but would probably need to be adjusted for other organisms.

**Figure 6:**
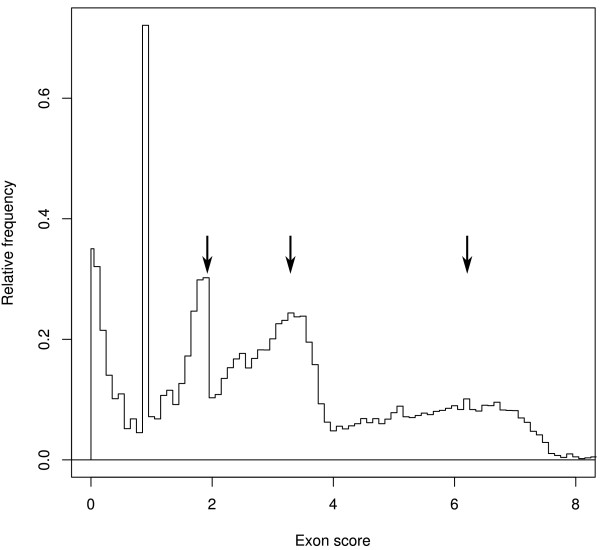
**Distribution of non-zero scores for *A. niger *exon models before selection of representatives.** The peaks marked by arrows near scores of 2, 3, and 6 arise from gene models with homology to 1, 2, or 3 known genes, respectively. In this sample, 44% of the exons had score 0.

Figure 6 illustrates the distribution of exon scores in pooled Augustus models from *A. niger*. Exon scores are affected mainly by the homology to known proteins of the peptides they encode and by the read-through ratios of their flanking introns. Imperfect models that have structural defects or are contradicted by the RNA-Seq evidence receive 0 scores. The scores of accepted models cover a broad range, reflecting the varying quality of the evidence for individual models.Overlaps are common in the pooled models from the Model Proliferation stage, and increase the size of the sets of models that must be compared to one another. Figure 3 illustrates the inputs to and outputs from model selection in part of a typical overlap group. Both the Splice-hinted models and the GeneMark models in this region contain gene predictions that fuse two genes, adding to model overlap. The Pooled Augustus models include various combinations of potential exons and introns, including several single-exon predictions. The selection procedure identifies the set of non-overlapping transcripts giving the highest total score, with credit for multiple independent predictions. There are three accepted models, matching manually curated models, in this region. All of the accepted models are present among the Pooled Augustus models; the leftmost one was also predicted by GeneMark, and the rightmost one by Splice-hinted Augustus. The representative models include a short imperfect model in the gap between the leftmost and central genes.

The sets of non-overlapping models produced by the selection procedure are usually small. In the Pooled Augustus models from *A. niger*, for example, the number of selected models per overlap group averaged 1.3; 82.7% of the overlap groups yielded a single selected model. At the other extreme, one group containing 112 overlapping models gave 11 selected models. Just 0.1% of the selected sets had more than 6 members. Nonetheless, the few large overlap groups consume most of the running time of the selection procedure.

### Computational load

The pipeline is computationally intensive and its execution time depends strongly on the processing power available to it. It needs at least 3 processors to run, and benefits from using more; the maximum number of processes that the pipeline can use is a configurable parameter. Hardware support for BLAST searches is especially helpful; the pipeline will optionally run Tera-BLAST software on local or remote DeCypher TimeLogic boards. On a workstation with 2.4 GHz Intel Xeon E5620 CPUs and remote access to a TimeLogic board, the pipeline took 10.1 h to predict the genes in the 20.3 MB *T. lanuginosus* genome. Without the TimeLogic board, the pipeline run took 19.6 h using 13 processes for parallel BLAST searching. Memory requirements are relatively modest; 2.5 GB of RAM per processor is sufficient for fungal genomes.

### Characteristics of accepted gene models

Table 3 summarizes the general features of the accepted gene models predicted by the SnowyOwl pipeline for three genomes: the ascomycetes *A. niger* strain ATCC 1015 [see Additional file 7] and *T. lanuginosus* [see Additional file 8], and the basidiomycete *P. chrysosporium* strain RP78 [see Additional file 9]. The SnowyOwl models can be viewed with Gbrowse at [29]. The average lengths of the genes, transcripts, exons, and introns are similar to those previously predicted for *A. niger* and *P. chrysosporium.* About 99% of the introns predicted by SnowyOwl have the GT-AG donor-acceptor consensus, with 1% showing GC-AG splice junctions, and 0.03% exhibiting the rare AT-AC junctions.

**Table 3:** Average dimensions of coding sequences in SnowyOwl accepted models and published models

	***A. niger***		***P. chrysosporium***		***T. lanuginosus***
	**(34.85 Mb)**		**(35.15 Mb)**		**(19.94 Mb)**
**Feature**	**SnowyOwl**	**Published **[30]**†**	**SnowyOwl**	**Published **[31]	**SnowyOwl**
Gene length, bp	1612.3	1708.9	1519.6	1667.0	1602.7
Transcript length, bp	1452.9	1484.7	1252.1	1356.7	1460.6
Protein length, aa	483.3	493.9	417.4	455.2	486.9
Exons per gene	3.1	3.4	5.2	5.9	3.1
Exon length, bp	466.4	442.1	239.0	233.6	468.8
Intron length, bp	75.4	95.1	63.1	64.2	67.2

†Updated January 2014.

### Characteristics of imperfect models

The largest class of imperfect models contains those predicting short proteins: fewer than 150 amino acids for monoexonic models with no homology support or fewer than 50 amino acids for models that contained introns or showed homology to known proteins (Table 4). Many of these short models are located adjacent to longer gene models and could result from short, nonfunctional ORFs inside the UTRs of the longer genes. Models with intron errors form the second most abundant class; they are located in genomic regions with significant RNA-Seq read coverage where Augustus and GeneMark failed to construct a model with introns that match the RNA-Seq spliced reads. Models that are inconsistent with RNA-Seq read coverage are also frequent; most often these models extend past the region of read coverage at one end, although some have internal coverage gaps. Representative models that have bad intron structure or cannot be translated into protein are relatively rare. Excessive length is the most frequent problem with intron structure. The unusually high number of representative models in *P. chrysosporium* that cannot be translated to protein are all incomplete gene predictions from GeneMark. The majority of untranslatable representative models in *T. lanuginosus* contain blocks of N introduced during scaffolding of the genome.

**Table 4:** Frequency of failure types in imperfect models

**Status**	***A. niger***	***P. chrysosporium***	***T. lanuginosus***
Bad intron structure	2	16	4
Cannot be translated to protein	5	120	39
Short predicted protein	4720	5977	3144
RNA-Seq coverage error	775	1859	560
RNA-Seq intron error	1923	1918	1229

### Comparisons of SnowyOwl models to previous gene predictions

**Figure 7:**
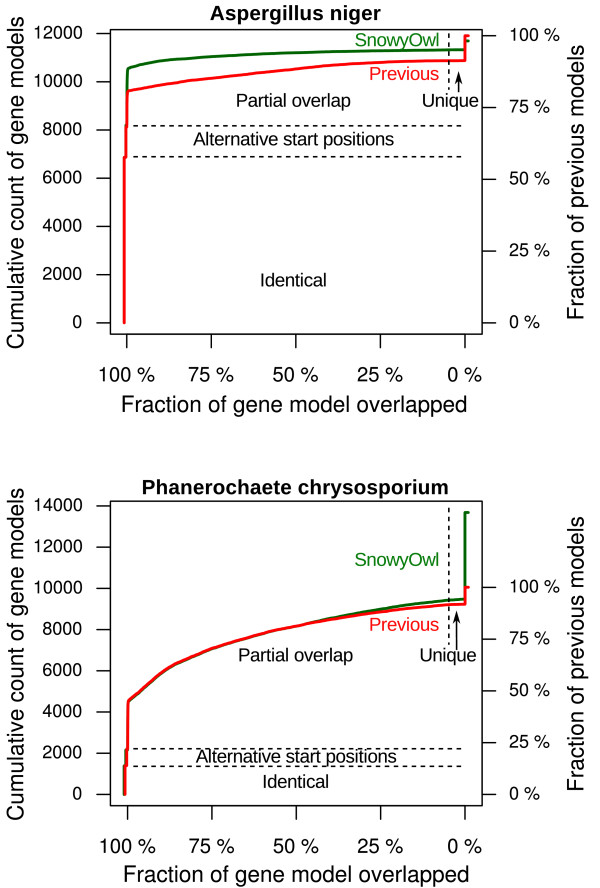
**Distribution of the degree of overlap between SnowyOwl and previous gene predictions in *A. niger *and *P. chrysosporium.*** Many SnowyOwl models, especially in *A. niger*, are identical to previous gene predictions or differ only at their start position, but there are some unique models in each set with less than 5% overlap in the other set, and numerous model pairs with intermediate levels of overlap. The higher numbers of gene models in the *P. chrysosporium* SnowyOwl set mainly result from predictions at locations where no gene was previously predicted. The counts for identical models and models differing only by alternative start positions are offset from 100% overlap for visibility.

Previously published gene predictions are available for *A. niger* and *P. chrysosporium*[30,31]. *Aspergillus niger* genes were predicted with Fgenesh, Fgenesh + [3], and Genewise [10] and representative models were selected on the basis of homology and EST support [30]; the updated gene model set v4.0 to which we compare SnowyOwl predictions was released in January 2014. The genes of *P. chrysosporium* were predicted with Genewise [10] and GrailEXP [48]. The overlap between SnowyOwl predictions and previous predictions is substantial in both genomes (Figure 7), indicating that SnowyOwl and the published models detect many of the same genes.

In *A. niger*, about 58% of the previously published predictions are identical to a SnowyOwl accepted gene model. Another 10% of the previous predictions are the same as a SnowyOwl prediction over much of their length but use a different start codon. Most of the remaining 32% of the models show some degree of overlap with members of the other set (Figure 7). A quarter of the SnowyOwl models overlap predictions in the other set by less than 100% but more than 50%; almost 22% overlap by 95% or more. The published models are longer on average than the SnowyOwl models, and some published models overlap two SnowyOwl models. Consequently the fraction of published models showing substantial overlap is lower: 14% overlap SnowyOwl models over more than 95% of their length and 20% over more than half their length. Some gene predictions are unique to one set or the other: 372 SnowyOwl predictions and 1038 previously published predictions have less than 5% overlap in the other set. Many (41%) of the published gene models that differ from a SnowyOwl prediction are incompatible with the RNA-Seq evidence (Table 5). Some of the published models contain structural errors; such defects are especially frequent in the models that overlap a SnowyOwl prediction by less than 5%.

**Table 5:** Comparison of published gene models to SnowyOwl predictions

	***A. niger***			***P. chrysosporium***		
**Similarity to SnowyOwl**	**Number**	Structural defects*	**Conflicts with RNA-Seq†**	**Number**	Structural defects*	**Conflicts with RNA-Seq†**
Identical	6868	0	0	1396	0	0
Identical except at start	1249	58 (4.6%)	438 (35.1%)	864	97 (11.2%)	216 (27.0%)
Different, > 5% overlap	2755	322 (11.7%)	1246 (45.2%)	6936	1924 (27.7%)	3750 (54.1%)
Different, < 5% overlap	1038	393 (37.9%)	376 (36.2%)	852	275 (32.3%)	442 (51.9%)
Total	11910	773 (6.5%)	2060 (17.3%)	10048	2296 (22.9%)	4408 (43.9%)

*Invalid coding sequences, short predicted proteins, introns with nonstandard donor-acceptor pairs.

†Predicted introns not matching read splice sites, models with discontinuous read coverage.

In *P. chrysosporium*, SnowyOwl predicted 13,683 genes, 36% more than the 10,048 predictions reported previously. A smaller fraction (ca. 12%) of the genes predicted in *P. chrysosporium* were identical in the SnowyOwl and previous prediction sets than in *A. niger*, but more of the non-identical models overlapped substantially. The model differences often involved the presence, absence, or position of small exons or introns, which are difficult to predict without RNA-Seq evidence; Figure 3 illustrates typical cases. The published gene models for *P. chrysosporium*, which date from the early days of fungal gene prediction, had a relatively high frequency of structural defects (Table 5). There were 5 times as many SnowyOwl as previous predictions that overlapped less than 5% with models in the other set. Some (12%) of these unique SnowyOwl predictions had homologs in the fungal RefSeq protein database supporting their validity.

Despite the generally higher quality of the SnowyOwl gene models, some of the previously published gene predictions were better than the corresponding SnowyOwl prediction or were missing from the SnowyOwl set. Applying the SnowyOwl scoring and selection procedure to the combined previous and SnowyOwl predictions gave totals of 11,894 accepted models in *A. niger* (6.0% unique to previous predictions; 25.3% unique to SnowyOwl) and 13,793 accepted models in *P. chrysosporium* (6.6% unique to previous predictions; 77.2% unique to SnowyOwl).

### Additional genomes analyzed with SnowyOwl

In addition to *T. lanuginosa*, we have successfully applied the SnowyOwl pipeline to 25 other novel fungal genomes. The resulting gene models are being publicly released in stages at [29].

## Discussion

The detailed information about transcribed gene sequences provided by RNA-Seq data allows a major advance in the accuracy of gene prediction. The SnowyOwl pipeline uses this information at several stages, from generating initial models through guiding gene prediction to scoring candidate models. The intron locations identified by spliced mapping of RNA-Seq reads are especially helpful in weeding out erroneous intron predictions by *ab initio* gene-predictors such as GeneMark and unhinted Augustus, errors that were frequent in the previous generation of fungal gene models [30,31]. Read coverage profiles reveal some gene models that are too long, including ones that concatenate genes. With the increased power to eliminate incorrect gene models provided by RNA-Seq, we can widen the range of candidate models generated by the *ab initio* predictors in order to increase prediction sensitivity. In addition to the gains in selectivity from RNA-Seq data, SnowyOwl increases the quality of its predictions by rigorously screening for correct intron and coding sequence structure.

We evaluated the performance of the SnowyOwl pipeline by comparing its predictions for the *Neurospora crassa* genome to independent, high-quality annotations. Over the whole genome, the sensitivity of the SnowyOwl gene predictions was 80.6% and their specificity was 65.3%. Both sensitivity and specificity depended on the level of RNA-Seq read coverage; among the genes with a read coverage depth of at least 0.5 reads per base, SnowyOwl’s prediction sensitivity was 88.4% and its specificity was 82.2%. The specificity of SnowyOwl is similar to those of GeneMark-ES and Augustus but its sensitivity is 11% higher on the *N. crassa* genes. We don’t have direct comparisons of SnowyOwl to other gene predictors, and no GASP-style competition has been held with a fungal genome. In the nematode *Caenorhabditis elegans*, the tested eukaryotic genome closest in size to a fungal genome, Augustus was among the most sensitive gene predictors in both the nGASP competition [22] (without RNA-Seq) and the RGASP competition [19] (with RNA-Seq). It appears that, within its domain of fungal genomes, SnowyOwl is at least as good as the best current gene prediction programs.

We also measured the ability of SnowyOwl to predict 5948 manually curated gene models from three other fungal genomes. This is not an independent test set, since the curator selecting those models relied on the same evidence as SnowyOwl uses. Nor is it unbiased, since most of the selected genes come from the subset with high read coverage where SnowyOwl is most effective. Nevertheless it was useful for checking how well SnowyOwl can use the RNA-Seq data to match the selections of a human expert in diverse genomes, for tuning the model-scoring routine, and for detecting losses of prediction sensitivity while streamlining the pipeline. The curated model sets also illustrate our progress from the previous generation of fungal gene models. SnowyOwl can predict 94% of the curated models from *A. niger* and *T. lanuginosus,* and 87% of those from *P. chrysosporium*. The numbers of these curated models in published gene sets for *A. niger* and *P. chrysosporium* are significantly less.

The pipeline’s design was streamlined by measuring the contribution of different components to the prediction of curated gene models in three test genomes. The key to obtaining adequate sensitivity proved to be generating a diverse array of candidate models by repeated Augustus runs with different inputs. Selectivity was gained by scoring the candidate models and choosing the best. Two computationally costly procedures that we expected to be rich sources of gene models, BLASTx search of the Uniprot database with all ORF sequences in the genome using TimeLogic® GeneDetective™ (Active Motif Inc., Carlsbad, CA) and Augustus runs guided by protein family profiles [11], were found to be redundant and eliminated from the pipeline during its streamlining.

Examining the false negatives and false positives in the SnowyOwl predictions relative to the curated models and the annotated *N. crassa* models reveals three areas in need of improvement: selecting start codons, predicting genes with low or no RNA-Seq coverage, and filtering short gene models. These all have high priority for future versions of SnowyOwl.

For 29% of the high-confidence annotated *N. crassa* models not exactly matched by a SnowyOwl prediction, there is a SnowyOwl prediction that differs only at its start; this fraction jumps to 48% in the models with adequate RNA-Seq coverage. The fractions of almost-matching predictions with misplaced start codons are even higher in the manually curated genes that were not predicted exactly by SnowyOwl: 68% in *A. niger*, 81% in *T. lanuginosus*, and 78% in *P. chrysosporium*. The affected genes have multiple ATG codons in frame with their ORF. The sequence contexts that favour recognition of start codons have been studied in mammalian and yeast genomes [49] and in one filamentous fungus [26]. Developing a context scoring method for potential start codons and using other markers such as signal peptides [50] could improve SnowyOwl’s prediction of CDS starts.

The sensitivity and specificity of SnowyOwl predictions for *N. crassa* genes with RNA-Seq read coverage depth below 0.5 were 45% and 27.5%, respectively, because insufficient RNA-Seq information was available to guide generation and selection of models for these genes. SnowyOwl was developed with an emphasis on well-expressed genes, and the best way to enhance its performance in a particular genome is to collect RNA-Seq data from a wide variety of growth conditions in order to increase the number of genes that are expressed. There will always be some genes whose transcripts remain unobserved, however, and better ways to handle these genes should be incorporated into SnowyOwl. The posterior probabilities estimated by Augustus might be useful guides for such genes.

Many of the false positive models from SnowyOwl predict short proteins (75% under 250 amino acids). The apparent specificity of Snowy Owl could be increased simply by raising the minimum length for predicted proteins, at the cost of missing more true genes. SnowyOwl currently uses homology to a database protein or presence of an intron as heuristics to rescue gene models with predicted proteins shorter than the minimum length. We need better ways to recognize genes encoding abnormally short proteins. Some of the false positive predictions are located just upstream of a true gene; they may arise from upstream ORFs (uORFs), which are common in fungi [51] and other eukaryotes [52].

The accuracy of SnowyOwl predictions may also be improved by better RNA-Seq technology and by taking technical limitations into account. At present SnowyOwl does not make use of strand-specific RNA-Seq because few strand-specific reads have been available. Strand-specific reads would allow transcription on the strand opposite to a gene model, which can inflate apparent model coverage and intron read-through, to be ignored and help separate overlapping transcripts on different strands [53]. Strand-specific RNA-Seq methods are becoming more standardized and commonly used [54], and it will be worthwhile to adapt SnowyOwl to take advantage of strand specificity. SnowyOwl assumes that all parts of a transcript are sampled equally, but the Illumina RNA-Seq library preparation method under-represents certain sequences [55]. Checking that any apparent gaps in read coverage are not artifacts of library bias before penalizing models for incomplete coverage would increase the accuracy of model scoring.

We developed SnowyOwl for use in fungal genomes and have only tested it with fungal genomes. It is highly configurable, however, and might be adapted for other types of genomes in need of a gene predictor.

## Conclusions

The SnowyOwl pipeline was developed to fill our need for rapid and accurate prediction of the genes in newly-sequenced fungal genomes using RNA-Seq data. Numerous programs are available to map RNA-Seq reads onto a genome sequence and to assemble the reads into potential transcripts. SnowyOwl takes the outputs of these programs as its inputs and packages the steps of training and running gene-predictors and selecting the best of their predictions.

SnowyOwl performs very well for genes that are represented in the available RNA-Seq reads; it predicted 88% of the *Neurospora crassa* genes that have adequate RNA-Seq coverage with 82% specificity. We have used it successfully in 26 novel fungal genomes.

SnowyOwl should be useful for the numerous fungal genomes currently being sequenced; it is freely available from Sourceforge for local installation and can also be accessed as a web service. The pipeline has been streamlined for computational efficiency and takes less than 24 hours to predict the genes in a typically-sized fungal genome. The gene model scoring parameters can be easily customized.

SnowyOwl can also be used to update older annotation that did not benefit from RNA-Seq information. It substantially improved upon previous gene predictions for *Aspergillus niger* and *Phanerochaete chrysosporium*. As well, SnowyOwl can incorporate the best of previous annotation into its predictions.

We intend to continue development of SnowyOwl as an open-source project to further improve its performance and to incorporate advances in RNA-Seq technology.

## Availability and requirements

In addition to the installable version of SnowyOwl, we have developed a web interface to the pipeline [see Additional file 10]. Registered collaborators at the University of Calgary’s Visual Genomics Centre can run the SnowyOwl pipeline remotely through this web interface; contact C. Sensen. Source code for the entire software package, including the Web interface, is available on Sourceforge.

**Project name:** SnowyOwl

**Project home page:**http://sourceforge.net/projects/snowyowl/

**Operating system(s):** Unix

**Programming language:** Python, PHP

**Other requirements:** Perl, Biopython, Augustus, GeneMark-ES, NCBI Blast+, exonerate, blat, samtools, tabix, cd-hit

**License:** FreeBSD

**Any restrictions to use by non-academics:** None

## Competing interests

The authors declare that they have no competing interests.

## Authors’ contributions

NO, PG, MD, and MA developed the model generation section of the pipeline, IR developed the model selection section, and OZ developed the web interface. JS, RN, OZ, and IR integrated and tested the pipeline. AT selected the curated models. IR drafted the manuscript. GB, CS, and AT conceived of the project, guided its progress, and edited the manuscript. All authors have read and approved the final manuscript.

## Supplementary Material

Additional file 1 **README.** Installation and usage information included with the SnowyOwl download package. Click here for file

Additional file 2 **CONFIG.template.** Default SnowyOwl configuration file with explanatory comments. Click here for file

Additional file 3 **Aspni curated models.** Manually curated gene models from *Aspergillus niger.* Click here for file

Additional file 4 **Thela curated models.** Manually curated gene models from *Thermomyces lanuginosus.* Click here for file

Additional file 5 **Phach curated models.** Manually curated gene models from *Phanerochaete chrysosporium.* Click here for file

Additional file 6 **Discarded pipeline components.** Descriptions of software modules used during development of the SnowyOwl pipeline but not included in the final version. Click here for file

Additional file 7 **Aspni accepted models.** SnowyOwl accepted models from *Aspergillus niger.* Click here for file

Additional file 8 **Thela accepted models.** SnowyOwl accepted models from *Thermomyces lanuginosus.* Click here for file

Additional file 9 **Phach accepted models.** SnowyOwl accepted models from *Phanerochaete chrysosporium.* Click here for file

Additional file 10 **Webapp README.** Installation and usage information included with the SnowyOwl web interface download package. Click here for file
