# Supplementary material for: SnowyOwl: accurate prediction of fungal genes by using RNA-Seq and homology information to select among ab initio models
Source: BMC Bioinformatics. 2014 Jul 1;15:229. doi: 10.1186/1471-2105-15-229 (PMC4084796; doi:10.1186/1471-2105-15-229)
Supplement: Additional file 6 — Discarded pipeline components. Descriptions of software modules used during development of the SnowyOwl pipeline but not included in the final version. [file 1471-2105-15-229-S6.pdf]

Discarded pipeline components

These modules were used during development of the SnowyOwl pipeline but not included in the final version.

Orthologs

The Uniprot-Swissprot database [1] was searched with the six-frame translation of the genomic sequence using DeCypher GeneDetective running on TimeLogic boards [2] to identify subsequences of the genome that encode homologs of known proteins. The structures of the transcripts corresponding to these subsequences were then refined using in-house scripts that took into account RNASeq coverage (for donor and acceptor sites) and potential frame shifts (genome assembly errors) identified using ESTScan [3]. The subset of the resulting gene models that were also found in at least one of GeneMark Models or Contig Models were used to train Augustus [4; 5].

Augustus Domains

Multiple sequence alignments of proteins representing 92 of the Glycoside Hydrolase families [6; 7] found in fungi were generated and then converted to block profiles as described in [8]. Augustus with the PPX extension [8], trained with the Consensus Training Set, was run on the entire genome for each profile. The resulting gene models were incorporated into the Pooled Augustus Models.

References

1 **Uniprot-Swissprot Database** [ftp://ftp.uniprot.org/pub/databases/uniprot/current_release/knowledgebase/complete/uniprot_sprot.fasta.gz]

2 **TimeLogic** [http://www.timelogic.com]

3 Lottaz C, Iseli C, Jongeneel CV, Bucher P: **Modeling sequencing errors by combining Hidden Markov models**. *Bioinformatics* 2003, **19 Suppl 2**:ii103-12.

4 Stanke M, Waack S: **Gene prediction with a hidden Markov model and a new intron submodel**. *Bioinformatics* 2003, **19 Suppl 2**:ii215-25.

5 Stanke M, Schoffmann O, Morgenstern B, Waack S: **Gene prediction in eukaryotes with a generalized hidden Markov model that uses hints from external sources**. *BMC Bioinformatics* 2006, **7**:62.
6 Henrissat B, Bairoch A: **Updating the sequence-based classification of glycosyl hydrolases**. *Biochem J* 1996, **316**:695-696.
7 **CAZy - GH** [http://www.cazy.org/Glycoside-Hydrolases.html]
8 Keller O, Kollmar M, Stanke M, Waack S: **A novel hybrid gene prediction method employing protein multiple sequence alignments**. *Bioinformatics* 2011, **27**:757-763.
